# Fluid Extracts

**Published:** 1868-06-15

**Authors:** H. D. Garrison

**Affiliations:** Chicago


### FLUID EXTRACTS.

CHICAGO, April 30, 1868.

*Editor Chicago Medical Journal:*

DEAR SIR:—An article in the last number of your JOURNAL entitled "Fluid Extracts," so fully opens up that subject, that a few observations upon it from a different standpoint, will probably not be uninteresting to many of your readers. All physicians — to their credit be it said — are exceedingly anxious to use the best possible remedies, and manufacturers, ever on the alert, often attempt to take advantage of this worthy zeal. One, having observed the interest manifested in those semi-sensational relations, of the wonderful effect of heat in causing decomposition of vegetable compounds, announces "Cold Expressed Fluid Extracts," with a great flourish, and many specious, with some good arguments. But, *nota bene*, the process is patented, which is a cheap, wholesale method of patenting the entire list of "Cold expressed fluid extracts." Now if the patentee can only succeed in driving the profession into the belief that his process makes better extracts than can be made otherwise, he has as perfect a monopoly in that line, as Dr. Payne has of his "family medicines." The fluid extracts to which you refer (Duffield's) are also made by a patented process, which, as before remarked, effectually patents the entire list. This view of the case, though worthy of serious consideration, is not the chief point to which I wish to direct special attention. Dr. Duffield's process, I believe, consists essentially in producing a vacuum in his percolator, and in his receiving vessel, and then allowing the menstruum to flow in, through a tube, upon the drug, whereby he gets a pressure upon his percolating menstruum of nearly fifteen pounds to the square inch, in addition to the gravity of the liquid. This method of rapidly filtering a liquid through a drug, if not new (as some broadly intimate), is certainly expeditious, which I believe is its chief merit. That it avoids heat (whether judiciously or not), is the bait offered the profession.

It is well known that many substances, though soluble to almost any extent in alcohol, are very *slowly* dissolved, *e. g.*, rosin, shellac, and most resins, as well as many alkaloids and neutral principles. Is it probable that such agents will be as thoroughly removed from the drug by a few minutes' rapid filtration, as by several days' contact with successive portions of fresh menstruum? Dr. Squibb found that when alcohol was slowly percolated through 16 oz. (Troy) of *finely powdered* cinchona bark, even the *fifth pint* of percolate (tincture) contained *two and a half per cent. of the entire alkaloids of the bark!* Could the first pint obtained be regarded as a perfect fluid extract?

But Mr. Duffield says his process removes the air, and thus allows the alcohol to permeate the drug more perfectly. Now when we consider that the medicinal virtues of plants reside almost entirely in the juice or sap which circulates through the lactiferous tubes which very freely anastomose with each other, and that this network is almost wholly broken up and destroyed by the process of fine grinding, and further, that alcohol is a very mobile liquid, having a powerful affinity for vegetable matter which causes it to permeate the finest capillary tubes with wonderful force, and the rapidity of magic, we utterly fail to see the necessity of a vacuum.

Suppose that some medicinal matter is lodged in unbroken tubes, cul-de-sac cells, etc., which is even freely soluble in alcohol, how is it to be carried out most perfectly? The liquid will go into these recesses *in vacuo*, or not quickly. When once in, all *vis a tergo* ceases. Being shielded by the walls of the tube or cell, it must remain comparatively quiet, despite the rapid currents outside. Diffusion alone can obtain the medicine from these recesses, and as this process is *slow*, it follows that gradual percolation, or even prolonged maceration, is more philosophic, than this rapid filtration.

This effort to totally avoid heat has unfortunately led its originator into another difficulty, which, if he has fully appreciated, I at least have not seen fairly stated, or even mentioned at all; viz.: the influence of a vacuum in promoting the decomposition of volatile bodies. Several mineral compounds are known to be so easily decomposed, that the presence or absence of pressure, or even of an atmosphere into which one of the constituents can diffuse itself, determines their stability, or decomposition. Following the cue of those who are so much exercised over the slight changes in composition which a moderate heat may effect, I may ask, if mineral bodies are thus separated, what must be the consequences of applying the same cause to those delicate compounds of highly volatile acids, alkaloids, æthers, oils, etc.

Aside from chemical dissolutions, a vacuum is a very prolific cause of physical separations. Liquids boil at a temperature of 145 degrees less *in vacuo* than in the open air; hence, subjecting a volatile substance to a vacuum at common temperatures (say 60° Fah.) amounts to the same thing, so far as dissipation of its substance is concerned, as heating it in the open air to 205 degrees (145+60=205). The vigorous pumping necessary to produce and sustain a vacuum, can not fail to remove considerable quantities of the most volatile parts of plants, as essential oils, ethers, etc.

I do not regard any one process as suitable for the production of all fluid extracts. Such a position is as untenable as a proposition to cure all diseases with one extract, or even the same disease under all kinds of circumstances and complications.

In the officinal processes (U. S. P. 1860), sixteen of the twenty-four fluid extracts admitted, are made by reserving (and never heating) an amount of the first tincture which passes by slow percolation, equal to three-fourths of the entire volume of extract to be made. The additional tincture obtained is evaporated by water bath to one fourth, and mixed with the reserved tincture. I modify this process, and as is believed, improve it, by reserving more of the first tincture, and evaporating the last tincture lower in a corresponding degree. With good, fresh drugs, ground to exactly the proper degree of fineness, and with alcohol of proper strength, failure to obtain uniformly good and reliable fluid extracts by this process implies profound ignorance of the subject, or unworthy motives.

H. D. GARRISON.

